# Extracellular Vesicles as Possible Plasma Markers and Mediators in Patients with Sepsis-Associated Delirium—A Pilot Study

**DOI:** 10.3390/ijms242115781

**Published:** 2023-10-30

**Authors:** Konstanze Plaschke, Thorsten Brenner, Mascha O. Fiedler, Tobias Hölle, Maik von der Forst, Robert Christian Wolf, Jürgen Kopitz, Johannes Gebert, Markus A. Weigand

**Affiliations:** 1 Department of Anesthesiology, University Hospital Heidelberg, Im Neuenheimer Feld 420, 69120 Heidelberg, Germany; thorsten.brenner@uk-essen.de (T.B.); mascha.fiedler@med.uni-heidelberg.de (M.O.F.); tobias.hoelle@med.uni-heidelberg.de (T.H.); maik.forst@med.uni-heidelberg.de (M.v.d.F.); 2 Department of Anesthesiology and Intensive Care Medicine, University Hospital Essen, University Duisburg-Essen, Hufelandstr. 55, 45147 Essen, Germany; 3 Center for Psychosocial Medicine, Department of General Psychiatry, University Hospital Heidelberg, Vossstraße 4, 69115 Heidelberg, Germany; christian.wolf@med.uni-heidelberg.de; 4 Department of Applied Tumor Biology, University Hospital Heidelberg, Im Neuenheimer Feld 224, 69120 Heidelberg, Germany; jkopitz@gmx.de (J.K.); johannes.gebert@med.uni-heidelberg.de (J.G.)

**Keywords:** plasma extracellular vesicles (EVs), sepsis-associated delirium (SAD), intensive care unit, proteomics

## Abstract

Patients with sepsis-associated delirium (SAD) show severe neurological impairment, often require an intensive care unit (ICU) stay and have a high risk of mortality. Hence, useful biomarkers for early detection of SAD are urgently needed. Extracellular vesicles (EVs) and their cargo are known to maintain normal physiology but also have been linked to numerous disease states. Here, we sought to identify differentially expressed proteins in plasma EVs from SAD patients as potential biomarkers for SAD. Plasma EVs from 11 SAD patients and 11 age-matched septic patients without delirium (non-SAD) were isolated by differential centrifugation, characterized by nanoparticle tracking analysis, transmission electron microscopy and Western blot analysis. Differential EV protein expression was determined by mass spectrometry and the resulting proteomes were characterized by Gene Ontology term and between-group statistics. As preliminary results because of the small group size, five distinct proteins showed significantly different expression pattern between SAD and non-SAD patients (*p* ≤ 0.05). In SAD patients, upregulated proteins included paraoxonase-1 (PON1), thrombospondin 1 (THBS1), and full fibrinogen gamma chain (FGG), whereas downregulated proteins comprised immunoglobulin (IgHV3) and complement subcomponent (C1QC). Thus, plasma EVs of SAD patients show significant changes in the expression of distinct proteins involved in immune system regulation and blood coagulation as well as in lipid metabolism in this pilot study. They might be a potential indicator for to the pathogenesis of SAD and thus warrant further examination as potential biomarkers, but further research is needed to expand on these findings in longitudinal study designs with larger samples and comprehensive polymodal data collection.

## 1. Introduction

Sepsis is a life-threatening organ dysfunction syndrome caused by the host’s inadequate responses to infectious agents [1]. Sepsis is one of the most common diseases worldwide with an estimation of about 50 million patients being affected every year and with approximately 20% mortality considered as sepsis-related. A study based in Germany found a sepsis incidence of 158 per 100,000 inhabitants [2]. 

Organ dysfunction is a major characteristic of sepsis, and, in particular, sepsis-induced brain dysfunction is highly prevalent with an early onset [3]. At a first acute phase, sepsis-induced brain dysfunction is often manifested as sepsis-associated delirium (SAD). SAD is mostly associated with changes in patients’ cognitive impairment and attention [4,5], and is further clinically characterized by changes in consciousness that range from confusion to delirium or even coma [6]. SAD affects up to 70% of patients with sepsis [7] and it often prolongs the stay in ICU and increases mortality among sepsis patients [8]. Up to now, however, effective diagnostic strategies in SAD are lacking [9,10]. Thus, new techniques or plasma markers for early diagnosis of SAD and cognitive impairment are urgently needed. As such an example for peripheral plasma markers, we propose the analysis of extracellular vesicles (EVs).

The generic term “EVs” describes a heterogeneous population of lipid bilayer-enclosed particles that are secreted by nearly all cells into the extracellular space [11,12]. According to their biogenesis and sizes, EVs can be classified into different EV subtypes: (i) EVs with a range of 30 to 150 nm originating from the endosomal pathway and (ii) plasma membrane-derived micro-vesicles (100 to 1000 nm) [13]. EVs are found in body fluids, such as saliva, urine, cerebrospinal fluid (CSF), and blood plasma. Their cargo consists of proteins, nucleic acids, carbohydrates, and lipids. According to their function, EVs are considered as key players for cell-to-cell communication by transferring a molecular message encoded by their cargo to recipient cells [13,14,15]. 

Recently, we investigated the role of EVs in animals after surgical stress [16] because surgical stress induces neuro-inflammation, thereby causing neurological symptoms ranging from sickness behavior to delirium and cognitive deterioration. Thus, we suggest that pro-inflammatory signaling must be operative between the peripheral and the central nervous system (CNS). It has been further shown, that EVs in the brain play a role in several CNS diseases, such as stroke, Parkinson’s disease, prion disease, Alzheimer’s disease, and as traumatic encephalopathy [17], pathologies of the brain which are associated with a neuro-inflammatory response.

As well known, neuro-inflammation is an innate immune response induced by the astroglia and microglia, which respond to a pro-inflammatory signal by the production of, chemokines, cytokines, reactive oxygen species and secondary messengers [18]. Various recent studies indicate that EVs may also play a pivotal role in the initiation and control of neuro-inflammation and therefore are considered as potential biomarkers [19,20]. In detail, EVs have recently generated immense interest following their discovery as mediators of intercellular communication by delivering functional proteins, mRNA transcripts, as well as miRNAs, to recipient cells. Although suggested to primarily serve as signaling organelles which also remove unwanted cellular components in the brain, accumulating evidence suggests that exosomes can also significantly contribute to the development of several neuro-pathologies as biomarkers. Biological markers specific for SAD could facilitate the development of potential therapies or monitor the disease process in more detail.

Various approaches have been applied to transfer anti-neuro-inflammatory compounds through the blood–brain barrier (BBB) to attenuate neuro-inflammatory reactions leading to brain pathologies [21]. The mechanisms underlying SAD seem to be complex and currently remain unclear. SAD has been attributed to a combination of neuro-inflammation, disturbances in brain perfusion, neuro-transmission, and also in BBB. Thus, the transport of pro-inflammatory EVs released during peripheral inflammation may also reach beyond the BBB and trigger neuro-inflammation via an activation of cerebral glia cells [22], eventually leading to SAD. 

To the best of our knowledge, there is only one review available investigating changes in EVs, summarizing the role of different microRNA biomarkers in SAD and describing the role of some proteins such as aquaporin 4 and galactin-9 [23]. In the present pilot study, however, the objective is addressed to the characterization of EVs as biomarker of SAD with a strong focus on proteins.

## 2. Results

### 2.1. Patient Characteristics

The results of the present pilot study can only be interpreted as orienting, and the *p*-value cut-off of 0.05 should be discussed with caution for clinical significance according to the small sample size.

We included 22 septic ICU patients in the present study: 16 male and 6 female patients with a mean age of 75.7 ± 6.2 years. Age did not significantly differ between SAD and non-SAD patients, *p* = 0.330 (two-sample *t*-test). Also, sex distribution did not significantly differ between the two groups, *p* = 0.987 (chi-squared test, see Table 1).

At the time of inclusion in this study, the patients had a RASS score between −2 and +1, and the Glasgow Coma scale was determined between 12 and 15 points with a body temperature of 36.7 ± 0.6 °C. Of 22 patients, 7 were invasively ventilated at the time point of blood plasma sampling for the EV analysis, 3 in the SAD group and 4 in the non-SAD group. The main reason for ICU stays of patients enrolled in this study was abdominal sepsis following surgery (*n* = 20) or urosepsis (*n* = 2). We did not exclude any patients from subsequent analyses. Finally, we examined the routine blood characteristics of ICU patients, from which no significant differences were observed between both groups (Table 2).

### 2.2. EV Isolation and Characterization

**Figure 1:**
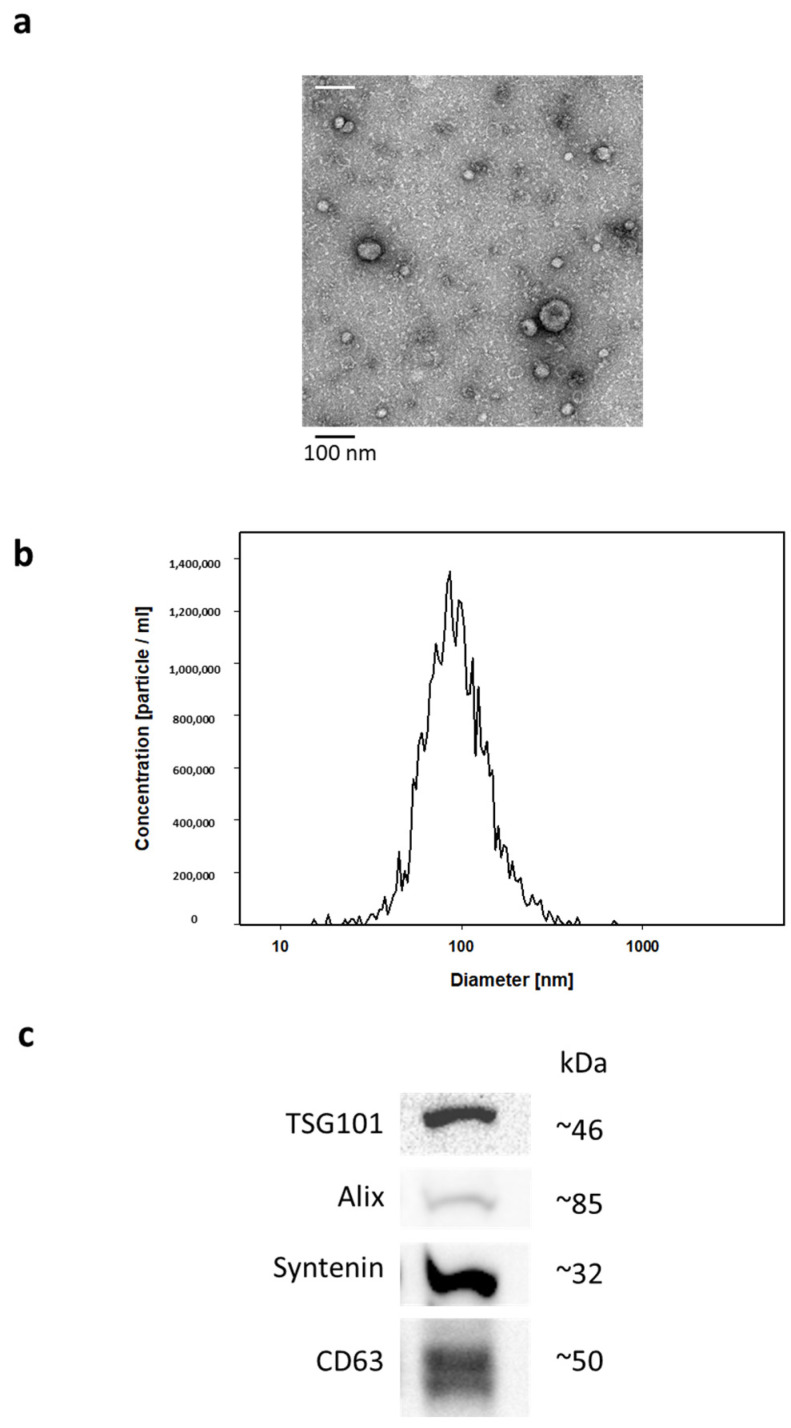
The identification and quality check of isolated EVs was performed using three different approaches which are exemplarily presented in Figure 1. (**a**) Transmission electron microscopy; (**b**) nanoparticle tracking analysis (NTA); (**c**) Western blotting.

The identification and quality check of isolated EVs was performed in compliance with the minimal information for studies of extracellular vesicles (MISEV) guidelines using three different approaches (Figure 1). First, transmission electron microscopy was used to visualize the sizes and structures of prepared EVs which were detected as a population of small (<150 nm) particles showing round and cup-shaped morphologies (Figure 1a). Second, nanoparticle tracking analysis (NTA) confirmed the observed size distributions average size of 95 nm and with a range of approximately 30–215 nm (Figure 1b). Finally, Western blot analysis validated the expression of EV specific markers of MISEV categories 1 and 2, like the cytosolic proteins TSG101, Alix and syntenin, as well as the transmembrane tetraspannin CD63 (Figure 1c). For more details, see Methods.

### 2.3. Proteomics Candidate Analysis

Mass spectrometry analysis of plasma EVs from septic ICU patients with and without SAD identified 592 proteins detected in at least one of the EV samples. Only including proteins that have non-zero intensity values in 75% of the samples in at least one of the conditions revealed a subset of five proteins that showed statistically significant differential expression in plasma EVs from patients with delirium (SAD) compared to patients without delirium (Table 3, *p* **). Paraoxonase-1 (PON1), known to be involved in reduction of poisoning agents. was found to be upregulated about 2.5-fold in SAD patients. Likewise, increased expression was also observed for Thrombospondin 1 (THBS1; 5.5-fold) and full Fibrinogen gamma chain (FGG; 1.4-fold), which indicate a more disturbed blood coagulation in SAD patients. In contrast, immunological markers such as immunoglobulin IgHV3 and complement factor C1QC showed significantly lower expression in plasma EVs of SAD patients (Table 3). Because of the small sample size, however, after FDR correction for multiple testing no significant changes were obtained. 

Taken together, plasma EVs of SAD patients show significant changes without FDR correction in the expression of distinct proteins involved in immune system regulation and blood coagulation, as well as in lipid metabolism. Whether they might be a potential indicator for the pathogenesis of SAD will be discussed in Section 3.

As indicated in Table 3 we also detected altered expression of several candidate proteins that showed an FDR-uncorrected value of *p* ≤ 0.1 (*p* *) and might represent additional potential blood markers for pathogenesis of SAD. The majority of these differentially expressed proteins represent different members of the large family of immunoglobulins (IgHV3OR16-12, IgLV3-9, Ig lambda, IgLC2, IgKV3D-11, IgKV2-40) which were reduced in SAD patients. A protein which is linked to lipid metabolism is the alpha-2-glycoprotein 1 (AZGP1), which is reduced in SAD patients. In addition, the acute phase reaction serum amyloid protein 1 (SAA1) and the C-reactive protein (CRP) seem to be implicated in the pathogenesis of SAD-delirium. 

The HbA1/HbA2 values were increased about by 2.5-fold in SAD patients. In addition to significantly changed THBS1 and FGG levels, the coagulation factor XIII A chain (F13A1) is another candidate marker whose expression was upregulated about by 2-fold in SAD patients. 

Further, the cadherin-like transmembrane glycoprotein desmoglein 1 (DSG1) was elevated by about 2.0–2.5-fold in SAD patients.

### 2.4. Results of GO Term Analysis

**Figure 2:**
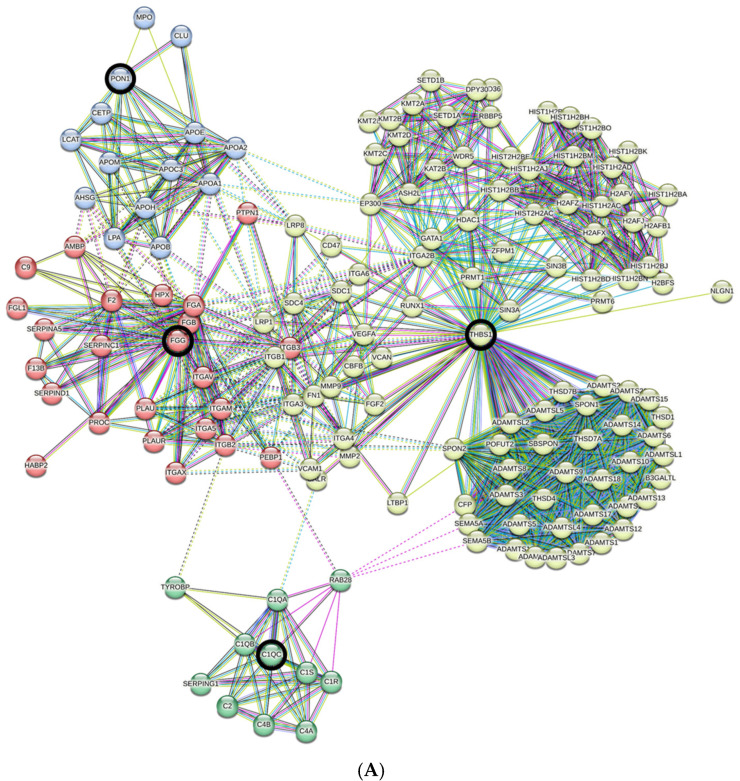

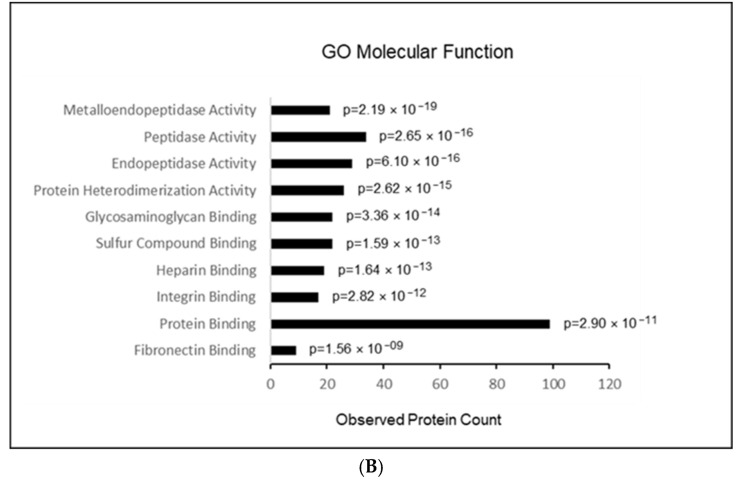

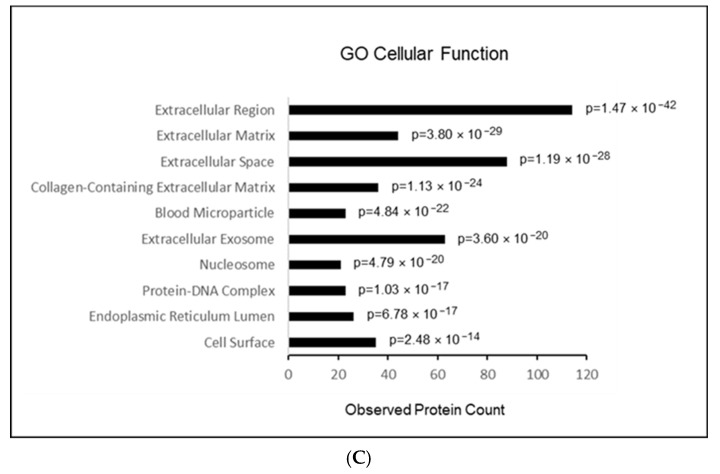
Proteomic data analysis: (**A**) Interaction network of four differentially expressed proteins (PON1, C1Q1, THBS1, FGG, red circles) generated by STRING v11.5. The connecting lines between protein nodes represent protein–protein interactions (PPI) with a maximal number of 500 interactions and based on an interaction score of highest confidence (0.9), a PPI enrichment *p*-value of <1 × 10^−16^ and a specified number of k-means clusters (*n* = 4). (**B**,**C**) Enrichment analysis of regulated proteins performed in STRING v11.5. Graphs showing most enriched Gene Ontology (GO) Molecular Functions, (**B**) Cellular Component, (**C**) among the top 10-regulated proteins with observed protein count in each category and calculated *p*-value corrected for multiple testing according to Benjamini and Hochberg [24].

Mass spectrometry analysis of plasma EVs from patients with and without SAD identified 592 proteins expressed in at least one of the EV samples (Table 3). Applying a cut-off value of *p* ≤ 0.05 revealed a subset of four non-immunoglobulin proteins that showed statistically significant differential expression in plasma EVs from SAD and non-SAD patients (Table 3). These proteins include the anti-oxidant and anti-inflammatory hydrolytic enzyme paraoxonase 1 (PON1), the complement cascade factor C1QC, the adhesive glycoprotein thrombospondin 1 (THBS1), and the blood-borne and coagulation cascade protein fibrinogen (FGG). With regard to known and predicted functionality and protein–protein interactions, the STRING database (http://string-db.org, version 11.5, STRING is a Core Data Resource as designated by Global Biodata Coalition and ELIXIR) represents a useful tool to provide some guidance on how these regulated proteins might interact directly or indirectly with themselves or with other proteins (see Section 4.10). We mapped together and visualized the four non-immunoglobulin regulated proteins by STRING in an interaction network extracted from the entire human genome (Figure 2A). The resulting network consisted of 150 nodes and 1291 edges. We used the GO analysis to support conclusions about the biology of a study system by identifying annotated functions that are overrepresented in subsets of genes of interest. Graphical visualizations of such GO term enrichment results are helpful for interpretation and avoid biases by presenting researchers with intuitive visual data summaries. A GO enrichment analysis (Figure 2B,C) showed strong enrichment in proteins involved in (endo)peptidase activity (*p* = 2 − 6 × 10^−16^) as well as binding of glycosaminoglycan (*p* = 3.36 × 10^−14^), heparin (*p* = 1.64 × 10^−13^), integrin (*p* = 2.82 × 10^−12^) and fibronectin (*p* = 1.56 × 10^−9^). The enrichment analysis also revealed that these regulated proteins can act in the extracellular region (*p* = 1.47 × 10^−42^), extracellular space (*p* = 1.19 × 10^−28^), extracellular matrix (*p* = 3.8 × 10^−29^) as well as in the extracellular exosome (*p* = 3.6 × 10^−20^).

In the Supplementary Materials, results of log2iBAQ heat map data (Figure S1) and PCA plot (Figure S2) are shown for SAD patients and the respective control group. 

## 3. Discussion

Several putative biomarkers related to the pathogenesis of delirium have been discussed in previous studies [25]. Surprisingly, research that considered blood markers of SAD beyond standard laboratory parameters has been scarce so far [23]. 

In this observational study we identified five abundant proteins (*p* ≤ 0.05) differentially expressed between sepsis patients with and without SAD, including paraoxonase-1 (PON1), immunoglobulin (IgHV3), complement subcomponent (C1QC), thrombospondin 1 (THBS1), and full fibrinogen gamma chain (FGG), in plasma EVs. In contrast, standard ICU blood characteristics did not differ significantly between the groups.

### 3.1. Paraoxonase-1 (PON1)

Several functions have been assigned to paraoxonase-1 (PON1) which we found upregulated in the present study. It has been shown to protect lipids against peroxidation and to prevent LDL oxidation; also a role in inflammation control was suggested [26]. Furthermore, PON1 and its isoforms seem to play an important role in drug metabolism (including a number of important pharmaceutical agents such as statins or glucuronide drugs) [26] and it has been associated with neurodegenerative diseases [27]. The enzyme is capable of hydrolyzing various types of substrate molecules, e.g., aryl esters, phospho-triesters, lactones and thio-lactones and thus may a function in the reduction of poisoning agents [26]. However, no physiological substrate of PON1 is known. Likewise the function of the enzyme in EVs is still completely unknown and should be an focus of further investigations.

Sepsis is a state of augmented oxidative stress and diminished anti-oxidant capacity. High density lipoprotein (HDL) particles were shown to possess anti-oxidant and anti-inflammatory properties, as well as PON1, which is an enzyme that is also protective against HDL oxidation. PON1 directly suppresses macrophage pro-inflammatory responses. These findings suggest that PON1 decreases sustained pro-inflammatory reactions, which subsequently can attenuate plaque progression. PON1 is also a major anti-atherosclerotic component of HDL [28]. The PON1 gene is activated by PPAR-γ, which increases synthesis and release of PON1enzyme from the liver, reducing atherosclerosis [29].

Interestingly, a very recent paper of our study group (Plaschke et al., 2023, accepted) addressing EVs in patients with Alzheimer’s disease—a disease with persistent cognitive dysfunction—showed a decrease in different apolipoproteins to approximately 50% as compared to age-related healthy volunteers in plasma EVs. Furthermore, HDL cholesterol levels have been shown to inversely correlate with brain Aβ deposits [30]. Conversely, low HDL cholesterol levels were associated with white matter changes, a higher probability of memory deficits, or a greater risk of dementia [31,32]. Many of these effects, such as anti-inflammatory and anti-oxidant effects, are mediated through HDL-protein cargo, which comprises many apolipoproteins (and several other proteins) that are associated with and form the HDL particles, extensively described in several studies [33,34]. It is possible that some comparable mechanisms in lipid metabolism might play a role in SAD patients as well as in Alzheimer’s patients (manuscript in press). 

Although other proteins, like the lipolysis-stimulating alpha-2-glycoprotein 1 (AZGP1) and the serum amyloid protein 1 (SAA1), which are associated with high density lipoproteins in blood plasma and secreted during the acute phase of inflammation protein and are also characteristic for lipid metabolism, were changed with less significance (*p* ≤ 0.1), they added impact to the importance of lipid metabolism in the pathogenesis of SAD. The same can be hypothesized for the cadherin-like transmembrane glycoprotein desmoglein 1 (DSG1) as well as desmocollin-1 (DSC1), which were increased (2.0–2.5-fold) in deliriant patients, respectively, whereas the galectin-3-binding protein (LGALS3BP)—a protein that in humans is encoded by the LGALS3BP gene—was reduced in SAD patients. Taken together, our measurement indicates a role for PON1 and associated changes in lipid metabolism in the pathogenesis of SAD.

### 3.2. Role of Immunological Markers 

It is now quite clear that systemic inflammation can also cause brain inflammation and neuro-inflammation is considered the main cause of neurological changes ranging from sickness behavior to delirium [35]. Consequently, the brain must be able to sense peripheral inflammation by communicating with the immune system. How the brain senses peripheral inflammation has been suggested as relating to several pathways. First, locally produced cytokines activate primary afferent nerves [36], giving a triggering signaling to the CNS [37,38]. Secondly, peripherally released pro-inflammatory cytokines may enter the brain through a disrupted blood–brain barrier (BBB) and therefore elicit neuro-inflammation [39]. Moreover, peripheral cytokines can gain access to the CNS via a specific BBB transport system [40]. 

In addition to these mechanisms, a novel mechanism has been suggested recently for peripheral signaling to the CNS, i.e., EVs are capable of passing the BBB. Therefore, EVs could be suggested as potential vesicles to transfer peripheral signaling molecules to induce delirium [41,42]. 

Delirium is an acute neuropsychiatric syndrome characterized by acute-onset cognitive deficits in perception and behavior. The pathophysiology of delirium is complex but inflammation seems to be a relevant precipitant factor, although it remains unclear how acute systemic inflammation induces the clinical alterations in delirium. Activated microglia is one key player responsible for an acute neuro-inflammatory reaction by exaggerated production of pro-inflammatory cytokines coupled with symptoms of sickness [43]. In Cerejeira’s work, changes in delirium underlie a more severe manifestation of sickness behavior with working memory deficits suggesting that inattention can be a clinical correlate with a pronounced neuro-inflammatory reaction. Our own published data revealed increased levels of pro-inflammatory cytokines and cortisol in plasma and cerebrospinal fluid of patients with delirium [44]. In addition, SAA and CRP might also play an important role [45], which is supported by our results showing altered expression (*p* ≤ 0.1) of both proteins in plasma EVs of SAD patients. However, to date the role of blood EVs in these processes has not been clearly resolved. 

Our data showed decreased levels of different immunoglobulins (Ig) in plasma EVs of SAD patients (Table 3). In conjunction with IgHV (*p* ≤ 0.05), albeit less significant (*p* ≤ 0.1), immunoglobulins (IgHV3OR16-12, IgLV3-9, Ig lambda, IgLC2, IgKV3D-11, IgKV2-40) support the link to immunological hypothesis in SAD. Interestingly, it is known that injection of non-specific immunoglobulin (IgG) into Rag/AD mice rescued microglial activation and improved plaque pathology, likely through binding to Ig receptors on microglia [46]. In addition, intravenous immunoglobulin was a classical immunotherapy and potentially reduces AD-type pathology by anti-Aβ, anti-tau, anti-inflammatory effects, and non-antibody-mediated effects [47,48], a chronic brain disease which is clinically characterized by cognitive dysfunction and has also been discussed with a link to the immunological hypothesis.

The complement system is another key component in the inflammatory response, which can be activated by the ‘DAMPs surge’ after surgery. Interestingly, although blood laboratory measurements of CRP (Table 2) showed no significant difference between both study groups, the iBAQ CRP showed a 1.8-fold increase in SAD patients of this study. The reason, therefore, is not known. We can only speculate that blood analysis is more of a snap-reading method with a great standard deviation in our study groups, whereas changes in proteins are more resistant to confounding factors. CRP is a biomarker that has been well established by studies involving various cohorts of delirious patients: thus it can be activated and regulated the classical complement pathway [49,50]. CRP is commonly used clinically as a blood biomarker also in delirium [51] and in postoperative patients or patients with multiple traumas characterized by an early complement component 3 (C3) activation, represented by plasma C3 depletion and upregulation of cleaved forms of C3 (including C3a and C3b) [52]. 

Further, C1q is also required for several complement-mediated activities. It is present in the neuropil, microglia, and a subset of interneurons in the brain and therefore could also be discussed as one pathogenetic factor of delirium. C1q, which is significantly reduced in the presented study, associates with the proenzymes C1r and C1s to yield C1, the first component of the serum complement system. An efficient activation of C1 takes place on interaction of the globular heads of C1q with the Fc regions of IgM or IgG antibody present in immune complexes [53]. 

Relating to the brain, complement is also involved in the elimination of neuronal synapses which is essential for adequate development, but can be harmful during aging or in cognitive disease [54]. Thus, the complement cascade not only provides protection from infection but can also mediate inflammation processes [54].

Taken together, our measurement supports the hypothesis that changes in immunological regulation are present in SAD. 

### 3.3. Role of Coagulation

Complement signaling closely interacts with the coagulation cascade, which also plays an important role in the inflammatory response, for instance after trauma, together with the release of DAMPs. Coagulation is also critically implicated in restoring tissue homeostasis [55,56]. 

Here we show marked changes in SAD patients: THBS1 and FGG were significantly increased. Albeit less significant, these deteriorations were accompanied by changes in HbA1/HbA2 and F13A1 (Table 3). 

The fibrinolytic system has well established immune consequences. It leads to formation of fibrin and initiates and resolves blood clotting. If fibrinogen enters the CNS parenchyma through a BBB opening, the insoluble fibrin becomes a strong immunogenic factor by binding to the CD11b I-domain of the CD11b/CD18 integrin receptor to further activate macrophages/resident microglia and to induce cognitive deficits [57]. Fibrinogen may also represent a valuable peripheral biomarker for identification of patients with increased risk for perioperative cognitive deteriorations, e.g., the elderly. Fibrin deposition has been implicated in several neurologic disorders, including traumatic brain injury and Alzheimer’s disease [58,59]. As is well known, fibrin is also a well-characterized pathologic hallmark of BBB disruption and therefore could represent a key mechanism for systemic and CNS inflammation and cognitive dysfunction after surgery [60]. 

Taken together, our measurement supports the hypothesis that dysregulation in blood coagulation is present in SAD.

### 3.4. Summary and Relevance

Regarding the possible effect of medication as a cofounder on our results, all ICU patients were treated according to the valid sepsis guidelines, including early source control, empiric antibiotic therapy, as well as supportive and adjunctive treatment measures. In case of SAD, symptomatic therapy with alpha-2-agonists (dexmedetomidin, clonidine) and/or neuroleptics was performed depending on the most prominent clinical symptoms. According to these therapies, it became clear that the influence of medication can be an important cofounder in the analysis of proteomic studies. However, it is not clear whether or not preoperative or intraoperative medication in addition to ICU medication may have an additional effect on iBAQ data. Unfortunately, because of the small group size, it is statistically not appropriate to do some subgroup analyses. In future, it should be the aim to carry out studies with many more patients to analyze all the above mentioned possible cofounders.

In comparing our results with the literature, the study of Sfera et al. [23] demonstrated an upregulated aquaporin-4 (AQP-4) protein originating in astrocytic end-feet which may be detectable in peripheral blood exosomes. Additionally, Sfera et al. discussed the role of galactin-9, CRYAB, A20 protein, vimentin in SAD. These proteins may also play, however partially, a role in immunological processes of SAD, as we also stated in the present study.

Plasma EVs of SAD patients show significant changes in the expression of distinct proteins involved in immune system regulation, blood coagulation, as well as in lipid metabolism. Thus, these peripheral biomarkers could be discussed as valuable tools for characterizing patients’ cognitive abilities [61] and further highlight the complex interactions between systemic inflammation and CNS pathology. As is well known, biomarkers are objective, quantifiable characteristics of biological processes. They should correlate with a patient’s experience in health and illness and—in the best case—they should correspond to patients’ clinical state. Particularly, in the cases of difficult diagnosis because of, e.g., unspecific clinical signs, they would offer additional support for the doctors’ decision regarding clinical diagnosis and treatment. For SAD, however, no specific evaluated biomarkers are known up to now. Therefore, we tried to find such markers in this hypothesis-generating study. The pilot character of this present study does not allow us to draw causal conclusions. However, we think it is important to investigate patients’ peripheral blood as an easily recoverable tool in order to draw conclusions about the cerebral state of analogous patients, as was done for non-ICU delirium in the past, and with the further aim completing the puzzle regarding the pathogenesis of SAD. Therefore, we used the proteomics method to find proteins and groups of proteins as risk factors differentiating SAD patients from non-SAD patients. However, because of multiple factors in the pathogenesis of delirium and especially of SAD, we can only speculate at this moment about the role of lipids, the immunological system and/or coagulation factors in this process.

The quest to find more specific diagnostic markers and to characterize pathogenetic changes in SAD with a greater number of patients and in more detail, however, will necessitate further studies to complete these complex processes. Biological markers with adequate sensitivity and specificity for SAD could finally decrease the length of hospital stay and lead to new treatments. Therefore, we see the clinical perspective for patients in a further development of biological markers for SAD, in order to help doctors underly their clinical diagnosis and to develop further diagnostic strategies for treatment of this disease.

For clinical relevance, one more possibility could be a current concept of biomarker-guided management for patients after further proteomic analysis. Proteomics is complex and reflects more than just the measurement of one single biomarker. However, after integration of proteomic markers into the context of a thorough clinical examination, standard blood parameters and a well-done risk stratification by clinical scores, proteomic biomarkers have great potential to improve the diagnostic and prognostic assessment of patients.

Limitations of this study include the modest sample size and the cross-sectional study design so that, at this stage, no inference can be made about protein changes over time, and about their predictive value in terms of disease progression or treatment response monitoring. Also, since we included ICU patients with sepsis only, we cannot draw any conclusions as to whether the proteomic changes may occur during the sepsis as compared to non-ICU patients. Proteomic studies with a limited sample size often lead to biased conclusions that cannot be repeated in other studies. 

Polypharmacy can also be discussed as one of the possible reasons for delirium, which was not analyzed in detail. Therefore, the delirium-specific increase in PON1 in SAD-patients due to polypharmacy cannot be excluded.

We also did not relate the proteomic findings to other biomarkers of the disease, e.g., SAA and CRP, which were changed, but failed significance levels of *p* ≤ 0.05, so that such associations have to be established by future studies, preferably in larger cohorts. Eventually, the absence of confirmation of iBAQ data through other techniques, such as ELISA or Western blot, could be considered as further potential limitations.

## 4. Materials and Methods

### 4.1. Study Design

This clinical observational cohort study was conceived as an exploratory pilot study without sample size calculation or power analysis. This study was conducted at the surgical ICU at the Department of Anesthesiology, Heidelberg University, Germany. The study was approved by the responsible Ethics Committee of the Medical Faculty of Heidelberg University (protocol #S-116/2017), registered in the German Clinical Trials Register (#DRKS00012346). The authors assert that all procedures contributing to this work complied with the ethical standards of the relevant national and institutional committees on human experimentation and with the Helsinki Declaration of 1975, as revised in 2008. The included non-delirious patients gave their written informed consent prior to inclusion in the study. Our delirious patients could not give consent themselves, but after setting up legal support, the legal representatives could sign the consent form.

### 4.2. Participants and Recruitment Process

This clinical study was performed under pandemic conditions, so that the recruitment of suitable patients was quite challenging and was performed as a hypothesis-generating study without sample size calculation. Related to the small sample size and to unjustified *p*-value for multiple testing, we can only consider this study as a pilot study with preliminary results. 

Once a patient on ICU was defined as septic (see definition below), we looked in the patients’ history for diagnosis for neurovascular or neurodegenerative disorders as an exclusion criterion, because these diseases are often related to persisting preoperative cognitive disturbances. In detail, palliative care, stroke, dementia or Parkinson’s disease were considered as exclusion criteria to eliminate signs of cognitive disturbances in patients’ history and to make the study group more consistent. Because of the small group size, no further inclusion or exclusion criteria relating to medication or artificial ventilation or other confounding factors were defined. Both study groups were distinguished by the condition ‘delirium’. Overall, *n* = 11 septic patients from ICU with delirium and *n* = 11 septic patients without delirium were included in this study

Thus, inclusion of septic patients in this study was based on required consent of ICU patients (with an age at admittance >18 years). Most included non-delirious patients gave their written informed consent prior to inclusion in the study. If our patients could not give consent themselves, legal support was set up and their relatives or the legal representatives signed the consent form. Therefore, we have on our ICU a standardized procedure for all patients who could give consent themselves, which is either given by their relatives or by a legal representative. 

The diagnosis ‘sepsis’ was made according to sepsis-3 criteria published in 2016 [62]. The SOFA score, which also assesses patient vigilance (GCS), plays a role in the sepsis-3 criteria. Vigilance is definitely a critical factor in delirious septic patients, which is why we also followed the current sepsis definition with the valid SOFA score. In brief, sepsis was defined as life-threatening organ dysfunction caused by a dysregulated host response to infection with an onset ≤24 h. Organ dysfunction was operationalized by an increase in the Sequential (Sepsis-related) Organ Failure Assessment (SOFA) score of 2 points or more. 

Delirium was determined using the German version of the ‘Intensive Care Delirium Screening Checklist’ (ICDSC) [63] and the ‘Confusion Assessment Method for the ICU’ (CAM-ICU) [64] at three times over 24 h by nursing personnel and/or by physicians, as also described in detail in our previous research [65]. If one of the tests was positive, the patient was defined as delirium-positive for this time point. 

### 4.3. Sample and Data Collection

Blood samples (4 mL) were collected in EDTA tubes at the time point of inclusion in the study as patients were defined as septic with or without delirium. Blood was centrifuged immediately and the remaining blood plasma was stored at −80 °C until EV isolation. At the time of blood sampling, several laboratory parameters were extracted from data routinely collected at the ICU. 

### 4.4. Clinical Data Analysis

To characterize the study cohort and the inter-group differences, means with standard deviations (SD) were reported for continuous patient data and absolute numbers with percentage proportions for categorical data. Differences between patient groups were calculated with a double-sided *t*-test for independent groups (for continuous data) and a chi-square test (exact Fisher test) for the evaluation of categorical data. Missing values were not imputed because of the small sample size. All calculations were made with IBM SPSS Statistics (version 27). A *p*-value ≤ 0.05 was considered statistically significant. 

### 4.5. Isolation and Characterization of EVs 

**Isolation:** Human plasma samples were thawed at 37 °C and diluted 1:5 (*v*/*v*) with PBS (Thermo Fisher Scientific, Waltham, MA, USA). Diluted samples were subjected to differential centrifugation (A 5000× *g*, 15 min, 4 °C; B. 10,000× *g*, 30 min, 4 °C). Cleared supernatants were transferred to sterile polycarbonate ultracentrifuge tubes and centrifuged at 120,000× *g* with k-factor 34.8 for 120 min at 4 °C (TLA-100.2, rotor 100.2, Beckmann-Coulter, Krefeld, Germany). After discarding the supernatant, each EV pellet was resuspended in 20 μL of PBS. Thereafter, the EV preparations were stored at −80 °C.

**Transmission electron microscopy:** The isolated EV samples were further diluted 1:20 (*v*/*v*) with PBS and transferred onto 100 mesh formvar-coated copper grids. Negative staining was performed with 2% aqueous uranyl acetate. Air-dried samples were visualized using a JEM 1400 transmission electron microscope at 80 KV (JOEL USA, Peabody, MA, USA) equipped with a Tietz 2K digital camera.

**Nanoparticle tracking:** Nanoparticle tracking analysis was performed according to the manufacturer’s instructions with the ZetaView PMX-220 TWIN Laser system and the software 8.05.05 SP2 (Particle Metrix, Inning, Germany). Measurements were performed in a dilution of 1:2000 (*v*/*v*) with PBS at 11 different positions. Settings for data acquisition were adjusted to a sensitivity of 95%, a shutter of 90, and a frame rate of 30 frames per second. 

**Protein extraction and Western blot:** Proteins were extracted in RIPA buffer (50 mM Tris–HCl pH 7.4, 150 mM NaCl, 1% Triton X-100, 1% Na-deoxycholate, 0.1% SDS, 0.1 mM CaCl_2_ and 0.01 mM MgCl_2_) supplemented with a complete Mini protease inhibitor cocktail inhibitor (Roche, Basel, Switzerland). Protein concentrations were measured following the manufacturer’s instructions by Bradford assay (Bio-Rad Laboratories, Hercules, CA, USA). Per sample, 50 μg (TSG101, Syntenin) or 30 μg (Alix, CD63) of protein were separated on 4–20% Bis-Tris gradient gels (Expedeon, Cambridge, UK) and thereafter blotted onto a nitrocellulose membrane (Life Technologies, Carlsbad, Germany). After blocking the membrane with 5% milk/TBST overnight at 4 °C, the following primary antibodies were used: anti-TSG101 (1:500, clone 4A10, Thermo Fisher Scientific, Waltham, MA, USA), anti-Alix (1:800, Q19, Santa Cruz, Heidelberg, Germany), anti-Syntenin (1:5000, clone ERP8102, Abcam, Cambridge, UK), and anti-CD63 (1:500, MX-49.129.5, Santa Cruz, Heidelberg, Germany). These primary antibodies were diluted in 5% milk/TBST and incubated with membranes overnight at 4 °C. The blots were washed with TBST and incubated with a sheep anti-mouse-IgG HRP (1:5000; GE-Healthcare, Chicago, IL, USA), donkey anti-goat-IgG HRP (1:1000; Santa Cruz, CA, USA) or a goat anti-rabbit-IgG HRP (1:2000; Promega, Madison, WI, USA) secondary antibody for 1 h at room temperature. Signals were detected using Western Lightning Plus ECL (Perkin Elmer, Waltham, MA, USA) and a ChemiDoc MP System (Bio-Rad Laboratories, Hercules, CA, USA).

### 4.6. Sample Preparation for MS Analysis

In order to remove reagents prior to tryptic digestion, the EV preparations protein was precipitated using a methanol–chloroform–water mixture. Precipitated protein samples (5 µg) were redissolved in 10 µL of 40 mM NH_4_HCO_3_ by 1 h incubation at 25 °C for at least 1 h. Thereafter, to completely reduce the disulfide bonds, 2 µL 10 mM DTT in 40 mM NH_4_HCO_3_ were added and incubated for 1 h at 45 °C. The resulting thiol groups were then alkylated by adding 1 µL 55 mM iodoacetamide (IAA) (30 min at 25 °C). Excess IAA was blocked by adding 2.5 µL 10 mM DTT for 15 min at 37 °C. Thereafter, tryptic digestion was performed with a trypsin:protein mass ratio of 1:100 overnight at 37 °C. Finally, the samples were stage tipped. Briefly, for each sample, 4 mg of a reverse phase material (OligoTM R3; Applied Biosystems, Foster City, CA, USA) slurry in ddH2O:acetonitrile (1:1, *v*:*v*) were retained in the pipette tip by a small portion of C18 material (Agilent Technologies, Santa Clara, CA, USA) conditioned by acetonitrile. Material was equilibrated with 2.5% formic acid. Samples were acidified by the addition of 10% formic acid to a final concentration of 2% and slowly passed through the material (with the addition of 2.5% formic acid to reach working volume). After five washing steps with 2.5% formic acid, elution was performed twice with 0.6% acetic acid in 80% acetonitrile. Eluates were dried completely in a vacuum centrifuge for mass spectrometric (MS) analysis.

### 4.7. MS Method Orbitrap Exploris 480

A LC-MS/MS analysis was carried out on an Ultimate 3000 UPLC system (Thermo Fisher Scientific, Darmstadt, Germany) directly connected to an Orbitrap Exploris 480 mass spectrometer for a total of 120 min. Peptides were desalted on a trapping cartridge (Acclaim PepMap300 C18, 5 µm, 300 Å wide pore; Thermo Fisher Scientific, Darmstadt, Germany) for 3 min using 30 μL/min flow of 0.05% TFA in water. The analytical multistep gradient (300 nL/min) was performed using a nanoEase MZ Peptide analytical column (300 Å, 1.7 µm, 75 µm × 200 mm, Waters GmbH, Eschborn, Germany) and using solvent A (0.1% formic acid in water) and solvent B (0.1% formic acid in acetonitrile). For 102 min the concentration of B was linearly ramped from 4% to 30%, followed by a quick ramp to 78%, then after two minutes the concentration of B was lowered to 2% and a 10 min equilibration step appended. Using a mass spectrometer, the eluting peptides were analyzed with data depend acquisition (DDA) mode. At 120 k resolution (380–1400 *m*/*z*, 300% AGC target, 45 ms maxIT), a full scan was followed by up to 2 s of MS/MS scans. Thereafter, peptide features were isolated with a window of 1.4 *m*/*z*, fragmented using 26% normalized collision energy (NCE). Fragment spectra were recorded at 15 k resolution (100% AGC target, 54 ms maxIT). Unassigned and singly charged eluting features were excluded from fragmentation and dynamic exclusion was set to 35 s.

### 4.8. Proteomic Data Analysis

Data analysis was carried out by MaxQuant (version 1.6.14.0) [66] using databases extracted from Uniprot.org under default settings (human containing 79,038 entries from 1 March 2022). Identification FDR cutoffs were 0.01 on peptide level and 0.01 on protein level. The match between runs (MBR) option was disabled.

LFQ quantification was carried out using a label free quantification approach based on the MaxLFQ algorithm [67]. For protein quantification, a minimum of two quantified peptides per protein was required. Thus, different values including iBAQ-and log2iBAQ values [68] were generated via MaxQuant. Protein groups marked as ‘Reverse’ and ‘Only identified by site’ were filtered out from the proteinGroups.txt file and subsequently formatted to XLSX. Respective heat maps and principle component analysis (PCA) were performed and, as examples, are shown as Figures S1 and S2 in the Supplementary Materials. 

### 4.9. EV Biomarker Candidate Data Analyses

All data including iBAQ and log2iBAQ were transferred into an Excel datasheet. Firstly, we determined the relative portion of each protein in relation to the sum of all proteins in the plasma probes. Secondly, we compared these relative data between patients with and without SAD using unpaired *t*-tests in order to see group differences without multiple testing (see Limitations). Only values that were at least 70% of the values in at least one of the conditions different of zero were included in the statistical analysis. *p*-values of ≤0.05 were considered as significant. Multiple testing was controlled by using an FDR of *p* ≤ 0.05.

### 4.10. GO Term Data Analysis

The global interaction network of regulated proteins was predicted in STRING v11.5 (available: at www.string-db.org, v11.5). Each protein–protein interaction (PPI) has a combined score (edge score), which represents the reliability of the interaction between proteins. For network visualization, the PPI interactions with a combined score (0: lowest confidence; 1: highest confidence) larger than 0.9 were used. In addition, enrichment analysis of regulated proteins was performed in STRING v11.5 for Gene Ontology Cellular Compartments and Molecular Function. Multiple testing was controlled by using an FDR of *p* ≤ 0.05. Visible clusters on PPI maps are assigned by color coding the nodes.

## 5. Conclusions

Keeping the above mentioned limitations of a hypothesis-generating study in mind, these preliminary results of our pilot study suggest specific proteomic differences of plasma EVs between septic patients with and without delirium. Therefore, we assume that proteomic load of EVs might contribute to pathogenesis of septic delirium. Considering the clinical relevance of septic delirium and the still limited opportunities for early diagnosis and reliable monitoring of disease progression and treatment effects, novel septic delirium diagnostic markers derived from peripheral biofluids, like blood, are urgently needed. This pilot study adds to that extant data by showing that plasma-derived EVs can be considered as a novel and rich source of potential disease biomarkers. However, further research is needed to expand on these findings in longitudinal study designs with larger samples and comprehensive polymodal data collection. 

## Figures and Tables

**Table 1:** ICU patient characteristics.

	SAD *N* = 11	Non-SAD *N* = 11	*p*-Value
Age	75.1 ± 6.9	76.3 ± 5.7	0.330
Sex (male:female)	8:3	8:3	0.987
Length of mechanical ventilation	24.2 ± 62.4	12.2 ± 12.2	0.269
Length of ICU stay	37.3 ± 63.1	22.8 ± 16.5	0.235
Length of hospital stay	68.1 ± 72.4	36.6 ± 27.7	0.096
RASS	0.0 ± 0.5	−0.3 ± 1.1	0.251
GCS	14.5 ± 0.7 *	13.4 ± 1.2	0.014
SOFA score	3.0 ± 2.8 *	6.6 ± 2.9	0.005
28-day mortality	1/11	2/11	0.876

Means ± standard deviation (SD). * *p* ≤ 0.05. Abbreviations: SAD: sepsis-associated delirium, ICU: intensive care unit, RASS: Richmond agitation scale, GSC: Glasgow coma scale, SOFA score: Sepsis-related organ failure assessment score.

**Table 2:** Results from blood gas analysis and conventional laboratory parameters.

	SAD	Non-SAD	*p*-Values
pH values	7.4 ± 0.1	7.4 ± 0.1	0.235
Blood glucose (mg/dL)	156.2 ± 68.4	129.4 ± 26.5	0.119
C-reactive protein (mg/L)	127.9 ± 80.5	148.2 ± 98.4	0.304
HCO_3_^−^ (mmol/L)	25.6 ± 4.7	23.3 ± 2.7	0.084
Platelets (/nL)	244.6 ± 129.8	187.2 ± 85.7	0.117
Creatinine (mg/dL)	1.3 ± 1.1	1.7 ± 1.0	0.176
Bilirubin (mg/dL)	0.9 ± 0.7	1.1 ± 1.3	0.325
Lactate (mg/dL)	11.5 ± 4.6	11.5 ± 3.2	0.489
Leucocytes (/nL)	11.5 ± 4.6	13.2 ± 7.3	0.259

Means ± standard deviation. Abbreviations: SAD: sepsis-associated delirium.

**Table 3:** Plasma EVs candidate proteins.

iBAQ-Values	SAD	Non-SAD	Ratio SAD/Non-SAD	*p*-Values
PON1	0.017 (0.010) **	0.007 (0.005)	2.5	0.005
IgHV3	0.074 (0.050) **	0.154 (0.094)	0.5	0.011
C1QC	0.512 (0.092) **	0.761 (0.357)	0.7	0.018
THBS1	0.0028 (0.004) **	0.0005 (0.0008)	5.5	0.026
FGG	6.422 (3.129) **	4.526 (1.924)	1.4	0.050
AZGP1	0.006 (0.006) *	0.016 (0.018)	0.4	0.059
SAA1	0.418 (0.483) *	0.164 (0.184)	2.6	0.059
HbA1/HbA2	0.513 (0.644) *	0.206 (0.143)	2.5	0.067
CRP	1.088 (0.976) *	0.589 (0.440)	1.8	0.067
HRNR	0.003 (0.005) *	0.0002 (0.0002)	9.8	0.069
IgHV3OR16-12	0.018 (0.016) *	0.031 (0.023)	0.6	0.072
IgLV3-9	0.005 (0.004) *	0.018 (0.016)	0.4	0.074
Ig lambda	0.213 (0.105) *	0.268 (0.016)	0.8	0.077
DSG1	0.004 (0.005) *	0.002 (0.002)	2.1	0.084
F13A1	0.005 (0.005) *	0.002 (0.002)	1.9	0.088
IgLC2	2.867 (1.235) *	3.702 (1.575)	0.8	0.090
DSC1	0.003 (0.003) *	0.001 (0.001)	2.2	0.092
LGALS3BP	0.106 (0.084) *	0.174 (0.144)	1.6	0.097
IgKV3D-11	0.498 (0.395) *	0.770 (0.547)	0.6	0.098
IgKV2-40	0.017 (0.020) *	0.031 (0.028)	0.5	0.098

iBAQ (Σintensity/#theoretical peptides) values are presented as means (SD). SAD: sepsis-associated delirium. Other abbreviations: see text. All *p*-values ** *p* ≤ 0.05, * *p* ≤ 0.1, ascending, cut off 75% in case of zero “0” values. For completeness, we also report findings at an FDR-uncorrected threshold of *p* ≤ 0.1.

## Data Availability

Original raw data is unavailable to share due to ethical considerations, and only pseudonymous data can be shared.

## Supplementary Materials

The following supporting information can be downloaded at: https://www.mdpi.com/article/10.3390/ijms242115781/s1.
