# Supplementary material for: Extracellular Vesicles as Possible Plasma Markers and Mediators in Patients with Sepsis-Associated Delirium—A Pilot Study
Source: Int J Mol Sci. 2023 Oct 30;24(21):15781. doi: 10.3390/ijms242115781 (PMC10649316; doi:10.3390/ijms242115781)
Supplement: Supplementary file 1 [file ijms-24-15781-s001.zip › ijms-2671918-supplementary.pdf]

International Journal of *Molecular Sciences*

MDPI

Supplemental Results

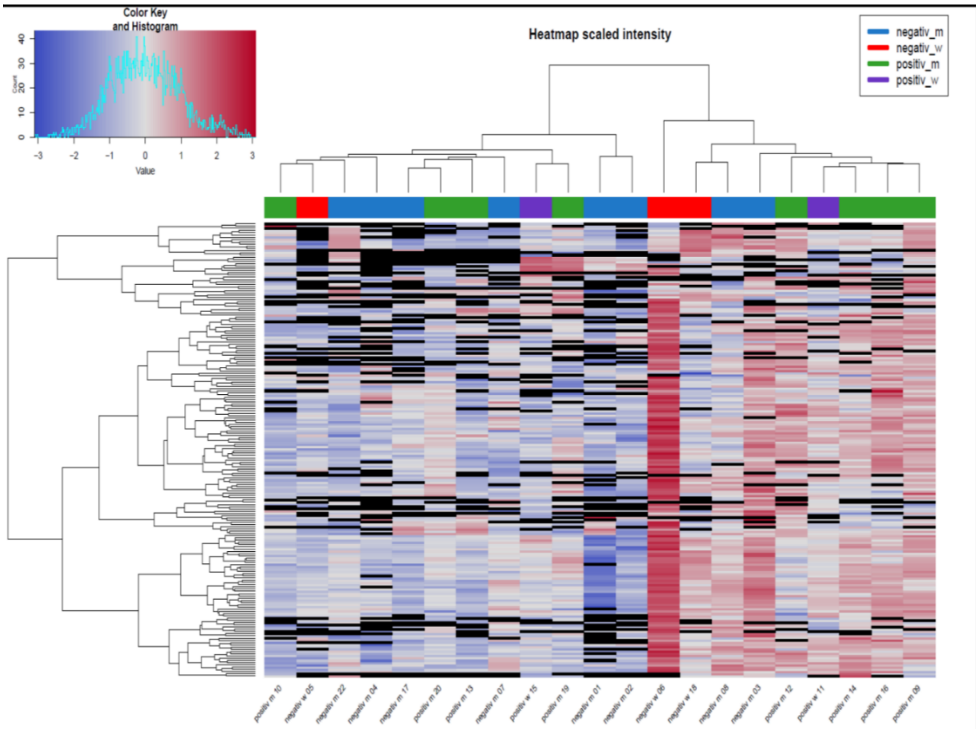

Figure S1. Log$_2$iBAQ_HeatmapScaled Intensity. Legend to Figure S1: Heatmap of scaled log2 transformed iBAQ data for patients with and without delirium and diffentiated for sex (m=male, w=female) and for SAD (positiv) and non-SAD (negativ) created in R with the function `heatmap.2` from the `gplots` package. The `dist` function was set to `euclidean` and `hclust` to `ward.D2`. Missing values are displayed in black.

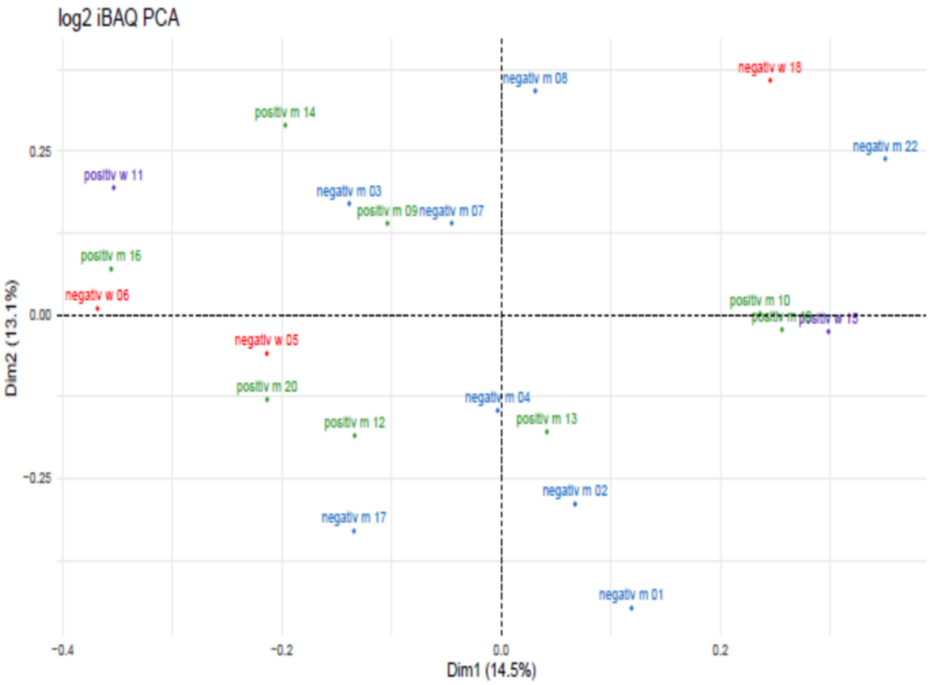

Figure S2. Log$_2$ iBAQ PCA. Legend to Figure S2: Principle component analysis (PCA) based on log2 transformed iBAQ data. Created in R based on the 'InDaPCA` for patients with and without delirium and differentiated for gender (m=male, f=female, SAD = positiv, non-SAD=negativ).
